# Surface Topography of Titanium Affects Their Osteogenic Potential through DNA Methylation

**DOI:** 10.3390/ijms22052406

**Published:** 2021-02-27

**Authors:** Young-Dan Cho, Woo-Jin Kim, Sungtae Kim, Young Ku, Hyun-Mo Ryoo

**Affiliations:** 1 Department of Periodontology, School of Dentistry and Dental Research Institute, Seoul National University, and Seoul National University Dental Hospital, Seoul 03080, Korea; cacodm1@snu.ac.kr (Y.-D.C.); kst72@snu.ac.kr (S.K.); guy@snu.ac.kr (Y.K.); 2 Department of Molecular Genetics, School of Dentistry and Dental Research Institute, Seoul National University, Seoul 08826, Korea; carpediemwj@snu.ac.kr

**Keywords:** surface topography, osteoblast differentiation, DNA methylation, epigenetics, gene expression

## Abstract

It is widely accepted that sandblasted/large-grit/acid-etched (SLA) surfaces of titanium (Ti) have a higher osteogenic potential than machined ones. However, most studies focused on differential gene expression without elucidating the underlying mechanism for this difference. The aim of this study was to evaluate how the surface roughness of dental Ti implants affects their osteogenic potential. Mouse preosteoblast MC3T3-E1 cells were seeded on machined and SLA Ti discs. The cellular activities of the discs were analyzed using confocal laser scanning microscopy, proliferation assays, and real-time polymerase chain reaction (PCR). DNA methylation was evaluated using a methylation-specific PCR. The cell morphology was slightly different between the two types of surfaces. While cellular proliferation was slightly greater on the machined surfaces, the osteogenic response of the SLA surfaces was superior, and they showed increased alkaline phosphatase (*Alp*) activity and higher bone marker gene expression levels (*Type I collagen*, *Alp*, and *osteocalcin*). The degree of DNA methylation on the *Alp* gene was lower on the SLA surfaces than on the machined surfaces. DNA methyltransferase inhibitor stimulated the *Alp* gene expression on the machined surfaces, similar to the SLA surfaces. The superior osteogenic potential of the SLA surfaces can be attributed to a different epigenetic landscape, specifically, the DNA methylation of *Alp* genes. This finding offers novel insights into epigenetics to supplement genetics and raises the possibility of using epidrugs as potential therapeutic targets to enhance osteogenesis on implant surfaces.

## 1. Introduction

Dental implants are widely accepted and used extensively to restore missing teeth. The key principle underlying the use of implants is osseointegration, i.e., the formation of a direct structural connection between the bone and the implant without any intervening soft tissue. The concept of osseointegration was defined by the Swedish orthopedic surgeon Per-Ingvar Brånemark after an accidental discovery in the 1950s [1], and dental implants were first used in patients in the mid-1960s. The interaction between osteoblasts and the Ti surface of implants is critical for ensuring early osseointegration and is thus the rate-determining step for reducing the implant treatment time. Numerous studies have been conducted on the implant surface modification to enhance osseointegration, resulting in a significant reduction of healing time and improvement in the implant success rate [2]. The majority of these studies concluded that rough implant surfaces aid osseointegration and that there is a significant relationship between the degree of surface roughness and the extent of osseointegration [3,4]. It is widely accepted that surfaces with moderately high roughness (R_a_, 1–2 μm) elicit a better osteogenic response than those with high roughness (R_a_ > 2 μm) [5,6], and surface roughness affects osteoblast gene expressions [7,8]. Various techniques to ensure that the implant surfaces exhibit the desired roughness were developed based on additive and subtractive methods. However, rough surfaces formed by additive methods, such as those involving the use of Ti or a hydroxyapatite plasma spray, were shown to increase the risk of peri-implantitis. Subtractive surfaces that exhibit moderately high roughness, including sandblasted/large-grit/acid-etched (SLA) surfaces and those formed using resorbable blast media and dual acid etching, were found to be successful in clinical studies [9,10].

Epigenetics is the study of molecular processes that affect the flow of genetic information between DNA sequences and gene expression patterns such as DNA methylation, post-translational modifications of histones, and RNA-associated silencing [11]. Among these, DNA methylation is a crucial epigenetic mark involving the transfer of methyl groups to the C5 position in CpG islands, and many studies have reported an association between gene expression and DNA methylation [12,13,14]. An in-depth understanding of the interactions between materials and cells is essential for the development of biomaterials suitable for clinical applications [15]. The materials act as a backbone for the cells to survive, allowing the cells organize into functional tissues [16]. Environmental cues, including material characteristics, such as their dimensions [17], topography [18,19], chemical composition, and physical properties [20], act as potent regulators of cellular activities, such as adhesion, proliferation, and differentiation [21]. It is well accepted that gene expression patterns also depend on factors other than the DNA sequence, that epigenetic factors regulate gene expression levels, and that the epigenetic landscape is affected by environmental factors [22]. Most previous studies on dental implants with surfaces with different levels of roughness have focused on differential gene expression without considering the role of epigenetics. Therefore, the aim of the present study was to elucidate the association between DNA methylation and gene expression and characterize the genetic and epigenetic patterns during osteogenesis on Ti surfaces with different levels of roughness.

## 2. Results

### 2.1. Characterization and Comparison of Ti Surfaces 

**Figure 1:**
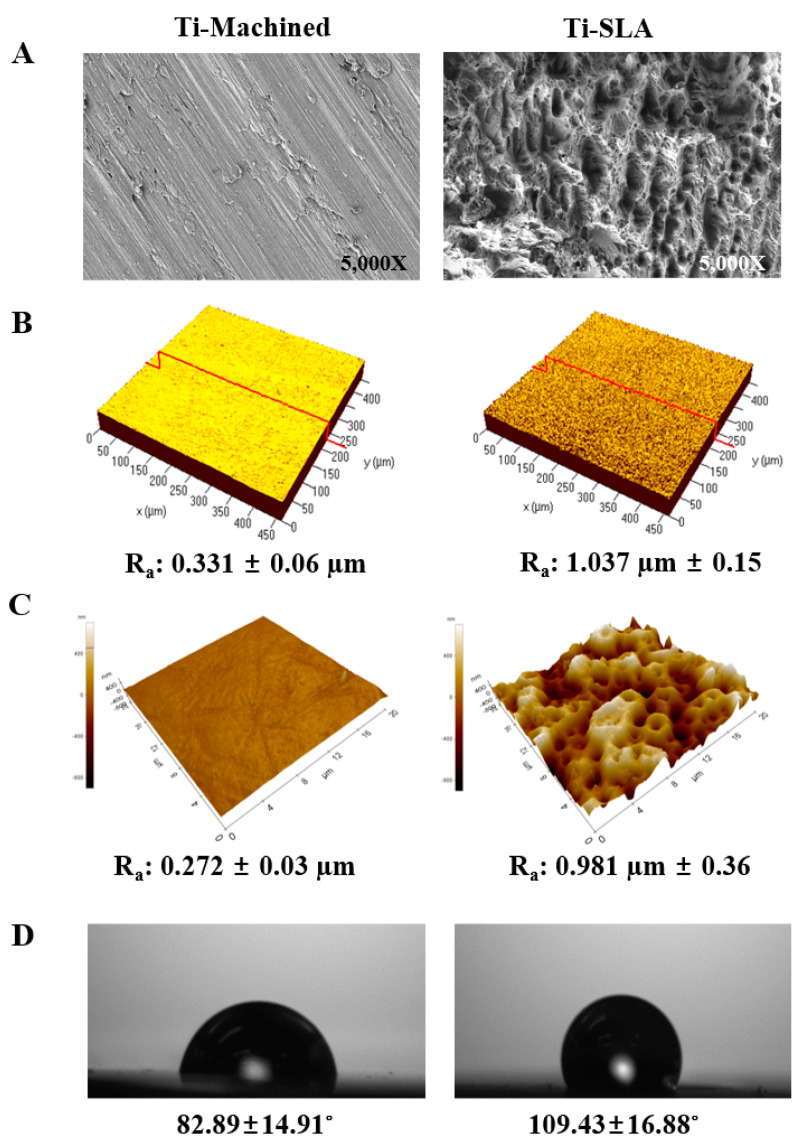
Characterization and comparison of Ti surfaces. (**A**) SEM images of two types of Ti surfaces investigated: Ti-M and Ti-S. Images are magnified 5000×. (**B**) CLM images indicating average surface roughness (R_a_). The red line is the cross-sectional line where the surface roughness parameter was measured. The value is expressed as mean ± standard deviation (SD) of three independent experiments. (**C**) AFM images indicating average surface roughness (R_a_). (**D**) Contact angle between the water drop and the substratum of discs. The value is expressed as mean ± SD of three independent experiments.

The surface morphologies of the machined (Ti-M) and SLA (Ti-S) surfaces were markedly different, as observed by scanning electron microscopy (SEM) (Figure 1A). A circular groove-like pattern was observed on the Ti-M surfaces owing to the machining process, whereas the Ti-S surfaces showed an irregular rough pattern with peaks and valleys. The roughness of the surfaces was measured as the average roughness (R_a_) by confocal laser microscopy (CLM) (Figure 1B). The R_a_ values of the Ti-M and Ti-S samples were 0.331 ± 0.06 µm and 1.037 ± 0.15 µm, respectively, and the atomic force microscopy (AFM) data (0.272 ± 0.03 µm and 0.981 ± 0.36 µm, respectively) were in agreement with the R_a_ values (Figure 1C). In addition, the contact angle was significantly higher in Ti-S (109.43° ± 16.88°) than Ti-M (82.89° ± 14.91°) (Figure 1D).

### 2.2. Cell Morphology

**Figure 2:**
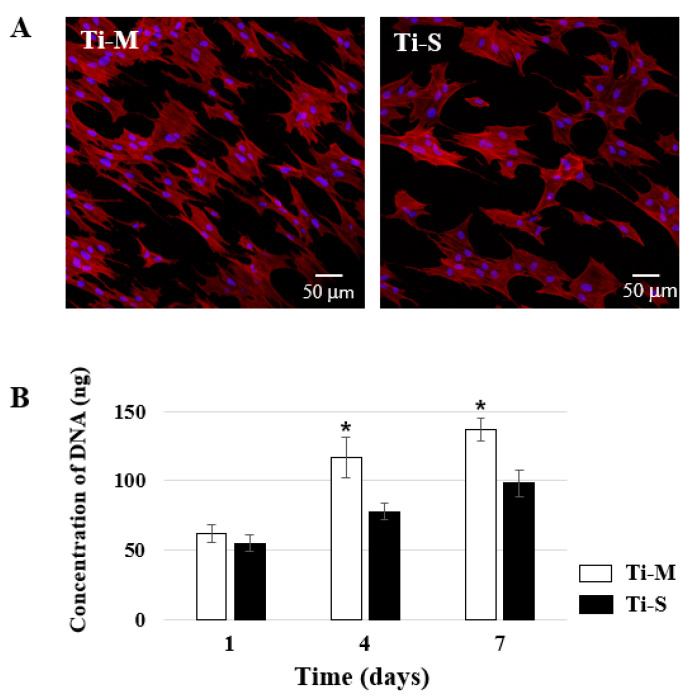
Cell morphology and proliferation. (**A**) Microscopic observation at 24 h after MC3T3-E1 cells were seeded on Ti discs. Blue and red fluorescence indicate the nucleus and cytoskeleton of the cell, respectively. Original magnification is 300× and scale bar = 50 μm. (**B**) PicoGreen^™^ assay (cell proliferation assay) of MC3T3-E1 cells at 1, 4, and 7 days after cell seeding. Data are expressed as mean ± SD of three independent experiments. * Significant differences between groups (*p* < 0.05).

After the MC3T3E1 cells were seeded on the discs for 24 h, cell morphology was examined by CLM. Blue and red fluorescence indicate the nucleus and cytoskeleton of the cell, respectively. The cells on the Ti-M samples exhibited a slightly more elongated shape that reflected the machining pattern, in contrast to the cells on the Ti-S samples. (Figure 2A). 

### 2.3. Cellular Proliferation 

The cells on the discs were harvested 1, 4 and 7 days after being seeded, and cellular proliferation was analyzed using the PicoGreen^™^ assay (Figure 2B). For both types of discs, it was observed that cell proliferation increased over the course of the experimental period until the 7th day. The cells proliferated more rapidly on the Ti-M discs than on the Ti-S discs. 

### 2.4. Osteoblast Differentiation

**Figure 3:**
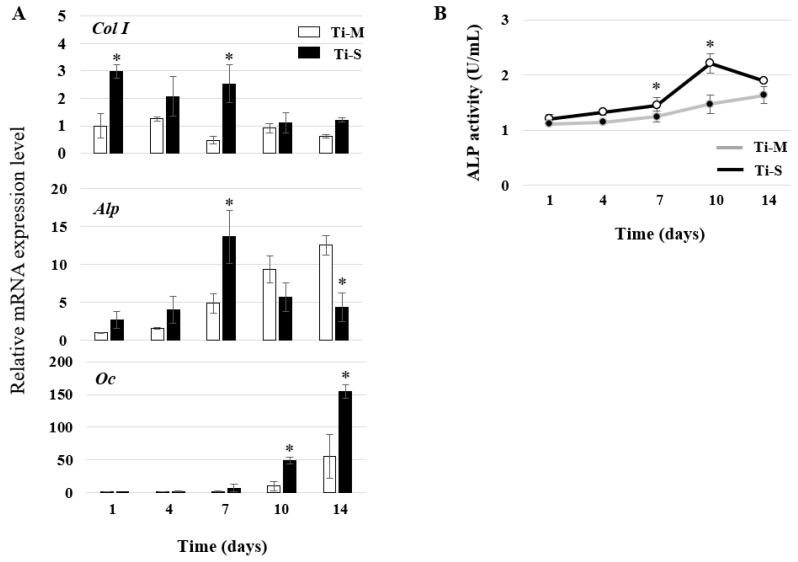
Comparison of osteogenic potential. (**A**) Real-time PCR. Representative bone marker gene expression 1, 4, 7, 10 and 14 days after osteogenic induction. Type I collagen (*Col I*), alkaline phosphatase (*Alp*), and osteocalcin (*Oc*) mRNA levels were determined. (**B**) ALP activity assay 1, 4, 7, 10, and 14 days after osteogenic induction. Data are expressed as mean ± SD of three independent experiments. * Significant differences between groups (*p* < 0.05).

The cells on the discs were harvested 1, 4, 7, 10 and 14 days after the induction of osteoblast differentiation in the osteogenic medium. Real-time polymerase chain reaction (PCR) was performed to evaluate the expression of the bone marker genes type I collagen (*Col I*), alkaline phosphatase (*Alp*), and osteocalcin (*Oc*) (Figure 3A). The expression of *Col I* was higher on the Ti-S discs than on the Ti-M discs. This was true for all the investigated time points, with the *Alp* expression on the Ti-S discs peaking on the seventh day before that on the Ti-M discs. There was little difference in the expression of *Oc* in the early stages until the seventh day. Thereafter, the increase was more pronounced on the Ti-S discs than on the Ti-M discs. The *Alp* activity assay showed well-differentiated patterns on both types of surfaces, but the osteoblast differentiation on the Ti-S discs was faster than that on the Ti-M discs (Figure 3B). 

### 2.5. DNA Methylation Pattern Analysis

**Figure 4:**
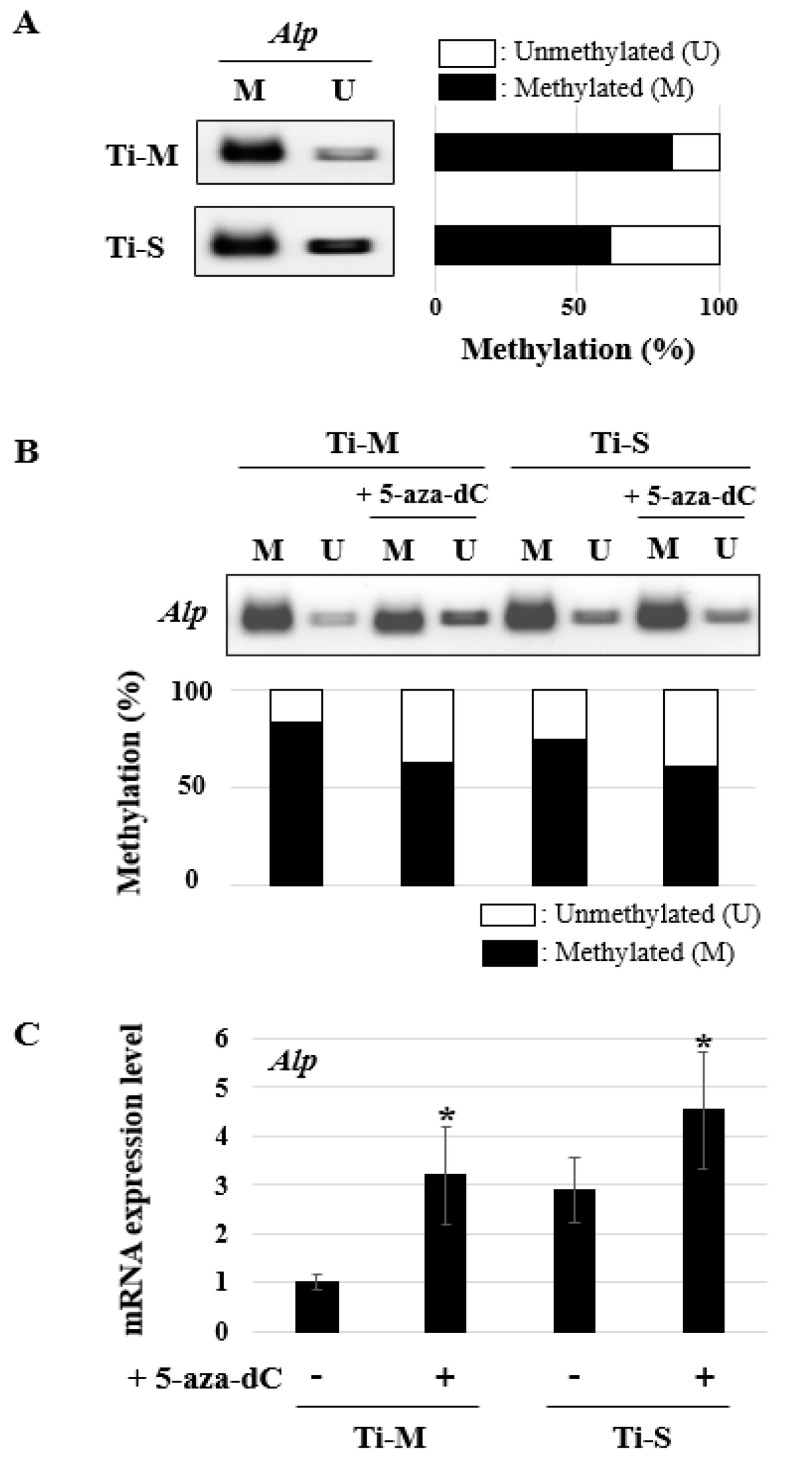
DNA methylation analysis. Methylation specific PCR (MSP) and real-time PCR were performed 7 days after osteogenic induction. (**A**,**B**) MSP was performed against *Alp*. M and U represent amplification of methylated and unmethylated portions, respectively. Quantification of MSP band density for methylated alleles (closed bars) and unmethylated alleles (open bars) is shown. (**C**) *Alp* mRNA expression level was determined using RT-PCR. * Significant differences between groups (*p* < 0.05).

We had reported previously that the *Alp* promoter region contains CpG islands and that the *Alp* gene expression level is inversely related to the DNA methylation pattern [23]. Based on these findings, the degrees of DNA methylation on Ti-M and Ti-S were evaluated 7 days after osteoblast differentiation because a significant difference in *Alp* gene expression was observed (Figure 3A). Through methylation specific PCR (MSP), it was found that the degree of DNA methylation in Ti-S was lower than that in the Ti-M discs (Figure 4A). To modify the degree of methylation, the cells on the Ti-M and Ti-S discs were treated with 5-aza-2′-deoxycytidine (5-aza-dC, 10 μM) for 24 h after being seeded, and then osteogenic induction was performed. The treatment with 5-aza-dC decreased DNA methylation on both discs (Figure 4B) and significantly stimulated the gene expression of *Alp* (Figure 4C). The *Alp* gene expression of the Ti-M surface with 5-aza-dC treatment was similar to the Ti-S surface, without 5-aza-dC treatment (Figure 4C). 

## 3. Discussion

Several studies on implant surfaces and osteoblast differentiation have focused on the differences in the osteogenic potential of surfaces based on gene expression levels, without fundamentally considering the underlying molecular mechanisms. At present, only a few studies have reported that the surface topography of implants can alter their cellular activities [21,24,25,26]. The primary hypothesis of this study was that the surface topography of Ti implants can modulate their cellular activities, such as cell morphology, proliferation, and differentiation, via differential gene expression based on epigenetics. This was derived from the evidence that the environment affects epigenetic changes which modulates gene expressions [27,28]. The main finding of this study is that different surface topographies induce epigenetic changes, especially DNA methylation, which regulate gene expression.

We determined the differences in the DNA methylation patterns in the promoter regions of the bone marker genes *Col I*, *Alp*, and *Oc* [29]. Among them, only the *Alp* gene showed differences in DNA methylation between the Ti-M and Ti-S surfaces (Figure 4A), and this manifested as a change in the *Alp* gene expression (Figure 3A). Furthermore, 5-aza-dC treatment decreased the methylation levels (Figure 4B) and stimulated the *Alp* gene expression (Figure 4C). This is because conditions that reduce DNA methylation allow transcription factors to combine, resulting in increased levels of gene expression. This conclusion agrees with our previous epigenetic studies [14,30,31,32], as well as those of other groups [33,34,35]. In addition, several studies have reported that the epigenetic basis of osteoblast differentiation and bone remodeling have the capacity to regulate cellular differentiation and the therapeutic potential in bone-related diseases [29,36,37,38,39]. 

Considering these data, we concluded that it is possible to modify the surfaces of dental implants with epidrugs to create an environment more favorable for osteogenesis. Lv et al. reported that TiO_2_ nanotubes promote the osteogenic differentiation of human adipose-derived stem cells by modulating the methylation level of histone H3 at lysine 4, in addition to that of DNA, in the promoter regions of the osteogenic genes *Runx2* and *Oc* [40]. Therefore, we attempted to find other epigenetic factors on histone modification; however, we did not observe any significant changes in the histone levels of the MC3T3-E1 cells (data not shown). 

In the field of tissue engineering, the triad of biomaterial, cells, and growth factors is intricately linked and thus essential for successful tissue or organ regeneration. In the past, it was considered that the biomaterial used merely served as the scaffold for supporting the cells and that the growth factors regulated the cellular activities via various signaling pathways. However, recent studies have shown that the biomaterial itself can modulate the cell responses independent of the growth factors [24,41]. Material The material characteristics that play an important role in cell response include surface energy and topography.

The surface energy of a material that measured indirectly by the contact angle is related with surface wettability and dependent on its surface chemistry [42]. Several studies have shown that cell adhesion is modulated by the surface energy of the underlying substrate, indicating a linear dependence on the surface hydrophilicity, which aids cell adhesion [43]. Likewise, changes in the surface topography in terms of roughness can increase the surface energy, thus enhancing hydrophilicity. Most studies showed that hydrophilic surfaces stimulate the early stages of cellular activities compared to hydrophobic surfaces; however, opposite results have also been reported [42,43,44]. In the present study, the contact angle in Ti-M was significantly lower than in Ti-S, indicating the high wettability of the Ti-M surface (Figure 1D). The cell morphologies of the two types of investigated surfaces were not significantly different (Figure 2A), however, cellular proliferation was higher in Ti-M (Figure 2B). The cells on the Ti-S discs exhibited cytoskeletons that were stretched in several directions in contrast to those on the Ti-M discs. A few studies reported that rough surfaces improve the entrapment of fibrin, resulting in better cell adhesion and proliferation [45,46]. However, the results of our study showed that cellular proliferation was higher on the Ti-M discs than that on the Ti-S discs, in agreement with several previous reports [41,47] and our previous data [48,49,50]. Although the reason for these contradicting results is not entirely clear, we think that several factors, such as the differences in the cell type, the cell seeding density, the surface processing procedure, and the proliferation assay method used, can explain the observed differences in the osteoblast proliferation on the Ti surfaces. In addition, we consider that surface wettability may act in the early stages such as cell attachment and proliferation, and epigenetic modifications based on surface roughness stimulate the gene expressions for cellular differentiation in the late stage. 

In this study, we found an important clue to explain why the SLA surfaces exhibited improved osteogenic characteristics compared with those of the machined surfaces from an epigenetic viewpoint, as well as a method for enhancing these characteristics. Additional in-depth research with more surfaces and the understanding of comprehensive epigenetic factors are needed to elucidate how the surface characteristics of dental implants affect their osteogenic properties through epigenetics. 

## 4. Materials and Methods

### 4.1. Materials

Commercially pure grade IV Ti discs with two types of surfaces, machined and SLA, were supplied by Osstem Implant Co., Ltd., Seoul, Korea. The discs were packaged and sterilized for experimental use and had a diameter of 25 mm and a thickness of 1 mm. The Ti-M discs were prepared through the machining process, and the Ti-S discs were prepared by sandblasting the Ti-M discs with 250–500-μm grit alumina particles and then acid etched with sulfuric acid.

### 4.2. Surface Analysis

The surface morphologies of the discs were observed using SEM (Carl Zeiss, Oberkochen, Germany). The surface topographies and roughness were observed using CLM (Carl Zeiss, Oberkochen, Germany) and AFM (XE-100, Park Systems Inc., Seoul, Korea). In the CLM, the red line is the cross-sectional line where the surface roughness parameter was measured. The average surface roughness (R_a_) was calculated based on the topography of the images and is represented by the mean ± SD of three independent experiments. For the measurement of the contact angle, distilled water was dropped onto the discs, and after 5 s, the contact angle was measured by a measuring device (Attension^®^ Theta Lite optical tensiometer, Biolin Scientific, Västra Frölunda, Sweden). Values represent the mean ± SD of three independent experiments. 

### 4.3. Cell Culture

Mouse preosteoblast MC3T3-E1 cells (ATCC, Manassas, VA, USA) were seeded on the discs and cultured in α-minimal essential medium (α-MEM) containing 10% fetal bovine serum and 1% penicillin/streptomycin (Hyclone, Logan, UT, USA). The cells were seeded on the discs at a density of 1 × 10^4^ cells/cm^2^ and incubated at 37 °C in humid air containing 5% CO_2_. To induce osteoblast differentiation, an osteogenic medium containing 10 mM β-glycerophosphate and 50 µg/mL ascorbic acid in α-MEM was used. The DNA methyltransferase inhibitor (DNMTi) 5-aza-dC (10 μM, Sigma-Aldrich, St. Louis, MO, USA) was used to treat the cells for 24 h to induce DNA methylation modifications. Three specimens were statically cultured per time interval, and three sets of cultures were examined for each experiment. 

### 4.4. Cell Morphological Observation

Twenty-four hours after seeding, the disc-adherent cells were fixed in 4% formaldehyde. ProLong^®^ Gold Antifade Mountant with 4′,6-diamidino-2-phenylindole (Invitrogen, Carlsbad, CA, USA) and Alexa Fluor 488 phalloidin (Invitrogen, Carlsbad, CA, USA) were used to detect the nucleus and the cytoskeleton, respectively. Fluorescence imaging was performed using CLM.

### 4.5. Cell Proliferation Assay

The cells on the discs were harvested 1, 4, and 7 days after cell seeding. The PicoGreen assay was performed with a Quant-It PicoGreen assay kit (Invitrogen, Carlsbad, CA, USA) to evaluate the cellular proliferation. The DNA content was determined by mixing with 100 μL of each sample and 100 μL of the PicoGreen reagent. Each sample was loaded in triplicate, and fluorescence was measured using a GloMax-Multi Detection System (Promega, Madison, WI, USA). The values shown represent the mean ± SD of three independent experiments (Supplemental Data A).

### 4.6. Reverse-Transcription PCR and Quantitative Real-Time PCR

The cells on the discs were harvested after 1, 4, 7, 10 and 14 days of osteoblast differentiation. RNA was isolated using the RNeasy^®^ mini kit (Qiagen, Valencia, CA, USA). The Primescript^TM^ RT reagent kit used for the reverse transcription was purchased from Takara Bio (Shiga, Japan). Quantitative real-time PCR was performed with primer sets for *Col I*, *Alp*, and *Oc* using a PowerUp™ SYBR™ Green Master Mix on a 7500 Real-Time PCR system (Applied Biosystems, Foster City, CA, USA). All samples were run in duplicate, and the relative expression levels were normalized with respect to glyceraldehyde-3-phosphate dehydrogenase. The values shown represent the mean ± SD of three independent experiments (Supplemental Data B).

### 4.7. ALP Activity Assay

The culture medium was collected after 1, 4, 7, 10 and 14 days of osteoblast differentiation. An ALP assay kit (Abcam, Cambridge, UK) was used according to the manufacturer’s instructions (Supplemental Data C).

### 4.8. Methylation Specific PCR (MSP)

To compare the DNA methylation pattern on the Ti-M and Ti-S surfaces, MC3T3-E1 cells were harvested after 7 days of the osteoblast differentiation. To evaluate the effect of DNMTi, the cells were treated with 5-aza-dC (10 μM) for 24 h before the osteoblast differentiation. Genomic DNA (gDNA) was isolated from the cells using a DNeasy Blood and Tissue kit, and the bisulfite conversion of the gDNA was performed using an EpiTect Fast DNA Bisulfite kit (Qiagen, Valencia, CA, USA). PCR was performed using the previously employed primers [14]. The intensity of the PCR band was measured using the software program Image J (National Institutes of Health, Bethesda, MD, USA).

### 4.9. Statistical Analysis 

All quantitative data are presented as the mean ± SD, and each experiment was performed at least three times. The results from one representative experiment are shown. Significant differences were analyzed using Student’s *t*-test. A value of *p* < 0.05 was considered statistically significant.

## 5. Conclusions

Within the limitations of this study, we concluded that the surface topography of Ti implants affects their osteogenic potential via epigenetic changes. The superior osteogenic potential of the SLA surfaces is attributed to a different epigenetic landscape, specifically, the DNA methylation of the *Alp* gene. This finding offers novel insights into epigenetics to supplement genetics and raises the possibility of using epidrugs as potential therapeutic targets to enhance osteogenesis on implant surfaces.

## Data Availability

Not applicable.

## Supplementary Materials

The following are available online at https://www.mdpi.com/1422-0067/22/5/2406/s1.
