# Supplementary material for: Surface Topography of Titanium Affects Their Osteogenic Potential through DNA Methylation
Source: Int J Mol Sci. 2021 Feb 27;22(5):2406. doi: 10.3390/ijms22052406 (PMC7957554; doi:10.3390/ijms22052406)

Experimental scheme

A Cell morphology was observed under CLM after 1 day after cell seeding, and PicoGreen™ assay was performed on days 1, 4, and 7 after cell seeding in normal medium.

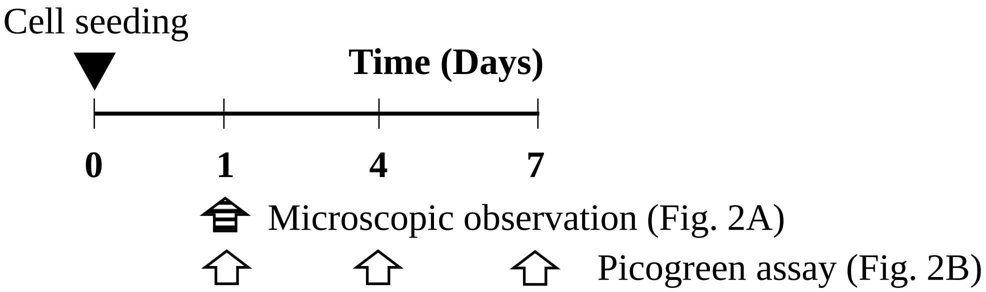

B Osteoblast differentiation was induced with osteogenic medium at 80% confluency after cell seeding. Real-time PCR and ALP activity assays were performed at days 1, 4, 7, 10, and 14 after osteogenic induction. Methylation specific PCR (MSP) was performed at day 7 after osteogenic induction

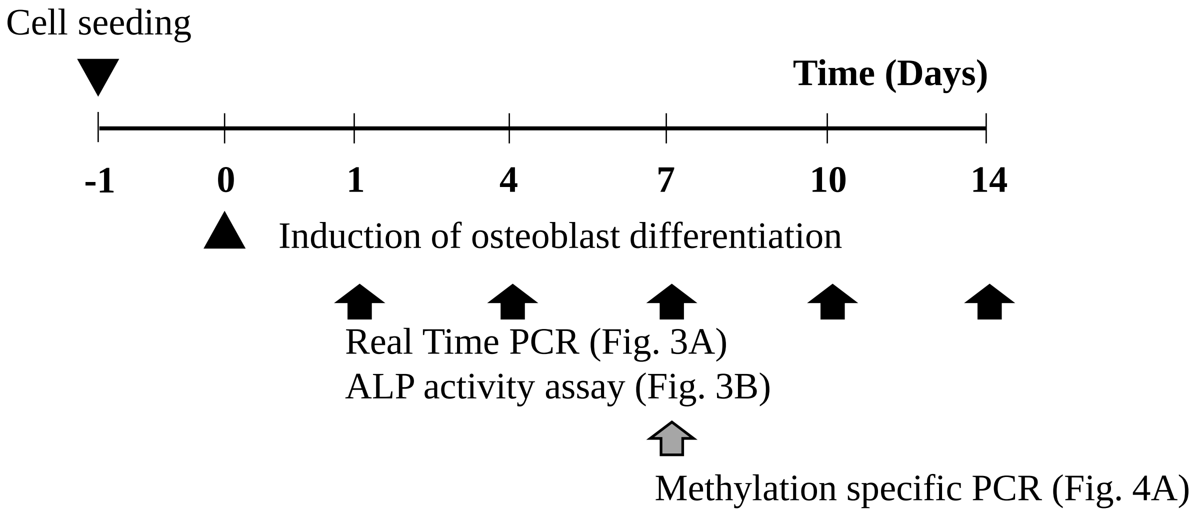

C After 1 day of cells seeding, 5-aza-dC was treated to cells for 24 hr, and MSP and real-time PCR were performed at day 7 after osteogenic induction.

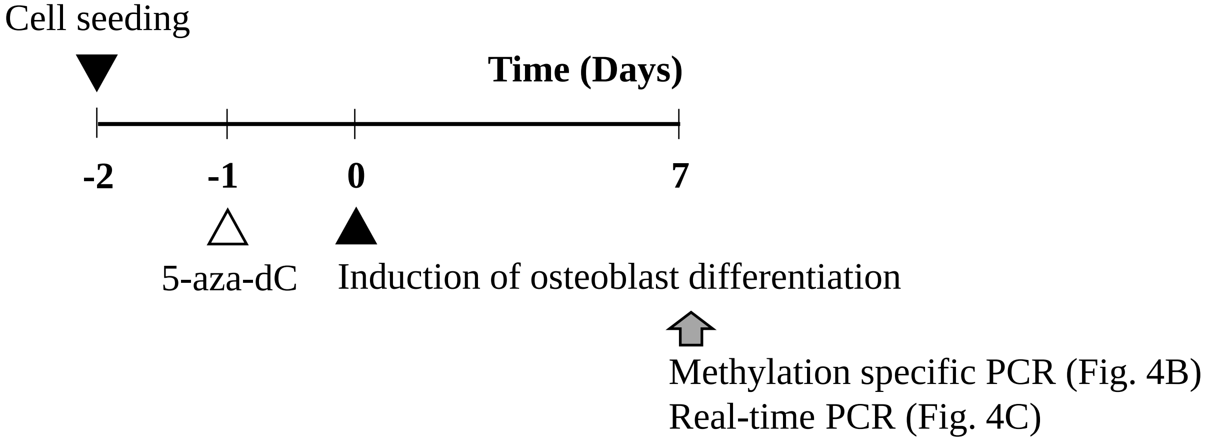

Supplement: Supplementary file 1 [file ijms-22-02406-s001.pdf]
